# Selective anti-tumor activity of glutathione-responsive abasic site trapping agent in anaplastic thyroid carcinoma

**DOI:** 10.1186/s12885-024-12511-3

**Published:** 2024-07-08

**Authors:** Jinyan Chai, Mengxue Su, Ruiguo Zhang, Ning Li, Yuanyuan Jia, Wei Zheng, Jian Tan, Qiang Jia, Huabing Sun, Zhaowei Meng

**Affiliations:** 1 Department of Nuclear Medicine, Tianjin Medical University General Hospital, Anshan Road No. 154, He ping District, Tianjin, 300052 P.R. China; 2 Tianjin Key Laboratory on Technologies Enabling Development of Clinical Therapeutics and Diagnostics, School of Pharmacy, The Province and Ministry Co-sponsored Collaborative Innovation Center for Medical Epigenetics, Tianjin Medical University, Tianjin, 300070 P. R. China

**Keywords:** Anaplastic thyroid carcinoma, Glutathione, Abasic sites, Apurinic/apyrimidinic endonuclease 1

## Abstract

Anaplastic thyroid carcinoma (ATC) is a rare but highly aggressive thyroid cancer with poor prognosis. Killing cancer cells by inducing DNA damage or blockage of DNA repair is a promising strategy for chemotherapy. It is reported that aldehyde-reactive alkoxyamines can capture the AP sites, one of the most common DNA lesions, and inhibit apurinic/apyrimidinic endonuclease 1(APE1)-mediated base excision repair (BER), leading to cell death. Whether this strategy can be employed for ATC treatment is rarely investigated. The aim of this study is to exploit GSH-responsive AP site capture reagent (AP probe-net), which responses to the elevated glutathione (GSH) levels in the tumor micro-environment (TME), releasing reactive alkoxyamine to trap AP sites and block the APE1-mediated BER for targeted anti-tumor activity against ATC. In vitro experiments, including MTT andγ-H2AX assays, demonstrate their selective cytotoxicity towards ATC cells over normal thyroid cells. Flow cytometry analysis suggests that AP probe-net arrests the cell cycle in the G2/M phase and induces apoptosis. Western blotting (WB) results show that the expression of apoptotic protein increased with the increased concentration of AP probe-net. Further in vivo experiments reveal that the AP probe-net has a good therapeutic effect on subcutaneous tumors of the ATC cells. In conclusion, taking advantage of the elevated GSH in TME, our study affords a new strategy for targeted chemotherapy of ATC with high selectivity and reduced adverse effects.

**Supplementary Information:**

The online version contains supplementary material available at 10.1186/s12885-024-12511-3.

## Introduction

Although anaplastic thyroid carcinoma (ATC) is a rare thyroid cancer (< 2%), ATC is extremely aggressive and lethal with a median survival of 4 months, and has high metastasis rates and refractoriness to conventional therapies, such as surgery, radioiodine therapy (RIT), external radiotherapy and chemotherapy [1]. Inhibitors targeting the PI3K/AKT/mTOR pathway work well for the treatment of other cancers, but the clinical outcomes for the treatment of ATC are disappointing, that limited benefit was observed for the patients [2]. There is no available therapy for prolonging the survival of patients with ATC at present. Hence, there is an urgent need to develop novel therapeutic strategies against ATC.

A large number of anti-tumor drugs damage DNA and promote apoptosis to kill cancer cells [3]. Among the drug-induced DNA lesions, abasic (apurinic/apyrimidinic, AP) sites are one of the most common types in cells, and can lead to more deleterious lesions such as DNA strand breaks [4, 5]. Consequently, the accumulation of AP sites is mutagenic and will yield cell death. Generally, more than 10,000 AP sites are produced daily in each cell, and can be efficiently repaired with the help of APE1 [6]. Whereas, aldehyde-reactive alkoxyamines capture AP sites, and inhibit the activity of APE1, the enzyme responsible for base excision repair (BER). Blocking the APE1-mediated repair of AP sites leads to cell death, and it is an actively investigated approach for treating cancers. However, unselective AP sites capture will kill all kinds of cells including normal cells, leading to potential adverse effects. Targeted anti-tumor agents that take advantage of the unique tumor micro-environment (TME) greatly reduce side effects on normal cells.

The selective release of alkoxyamines by an abundant biomolecule in cancer cells offers a solution for this challenge. We describe the use of glutathione (GSH) for this purpose, which is present at elevated levels (10 mM) in various cancer cells [7]. GSH acts as the critical antioxidant to modulate the cellular redox state and is also a strong nucleophile. GSH selectively recognizes 2,4-dinitrobenzene sulfonamides (DNS) to release SO_2_ and amines [8]. Benefiting from this strategy, we developed GSH-responsive AP sites capture reagent (AP probe-net), DNS caged coumarin-based alkoxyamine, showing targeted anti-tumor activity [9, 10]. The agent can be selectively activated by GSH to generate reactive alkoxyamine that traps AP sites in cancer cells. The GSH-responsive AP probe-net blocks the APE1 activity, thereby inducing cell death.

This study is to explore a GSH-responsive AP probe-net, which can be activated by the abundant GSH in ATC cells to generate reactive alkoxyamine, and the formed alkoxyamine can trap AP sites and block the APE1 activity, leading to ATC cell death but less adverse effects towards normal cells.

## Materials and methods

### Materials

3-(4,5-dimethylthiazol-2-yl)-2,5-diphenyltetrazolium bromide (MTT) was obtained from Beijing Dingguo Changsheng Biology Technology Co., Ltd. (Beijing, China). Roswell Park Memorial Institute 1640 (RMPI 1640) medium, fetal bovine serum (FBS), 0.25% Trypsin-EDTA, and penicillin streptomycin were purchased from Gibco (Thermo Fischer Scientific, Waltham, MA). Dimethyl sulfoxide (DMSO) was purchased from Concord Technology (Tianjin, China). Nthy-ori 3 − 1 cells, H1299 cells and ATC cells (THJ-16T and CAL-62) were provided by institute of Cell Biology (Shanghai, China). The deionized water used in this study was acquired from a Milli-Q synthesis system (Millipore, Billerica, MA). All reagents in this work were of analytical grade.

### Fluorescence imaging of GSH

Normal thyroid Nthy-ori 3 − 1 cells were used as negative control, lung cancer H1299 cells were used as positive control, and two kinds of ATC cells were treated with AP probe-net to evaluate GSH levels. First, the above four cell lines were inoculated in a confocal culture dish at a density of 1 × 10^5^ per well and cultured in incubators (37℃, 5% CO_2_) for 24 h. Then, the cell medium was replaced, and 10 µM of AP probe-net was added and incubated for 1 h. Finally, after washing twice with PBS buffer, the cells were photographed with fluorescence microscope (Leica DMI 3000B Germany, Coumarin-channel: Ex 405 nm/Em 461 nm).

### MTT cytotoxicity studies

The in vitro cytotoxicity of AP probe-net against ATC cells (THJ-16T and CAL-62) and normal thyroid cells (Nthy-ori 3 − 1) were evaluated by the MTT assay. Firstly, the above three kinds of cells were transplanted into different 96-well cell culture plates at a density of 1.5 × 10^4^ cells per well (in triplicate) and cultured in incubators (37℃, 5% CO_2_) for 24 h. Next, the cell medium was replaced, and 100 µL of different doses of AP probe-net (final concentration: 100, 75, 50, 25, 12.5, 6.25, 3.12 and 1.56 µM) were added and incubated for another 24 h and 48 h, respectively. The cell medium was replaced again, and 10 µL of MTT (5 mg/mL) was added and incubated for 4 h to generate the MTT-formazan. Finally, 150 µL of DMSO was added, and the absorbance was measured at 492 nm by a microplate reader (Bio-Tek, USA). The viability of the cells was calculated as a percentage of viability of the AP probe-net treated cells compared to the control.

### Flow cytometry analysis

Cells for apoptosis analysis by flow cytometry were seeded in six-well plates at a density of 3 × 10^5^ per well (in triplicate) and cultured for 24 h. And then distinct doses of AP probe-net (5, 20 µM) and DMSO as control were added. Then, the cells were digested with pancreatic enzyme without EDTA for 1 ~ 2 min. Upon terminating digestion, the cells were collected in the corresponding centrifuge tubes by a centrifugation (1000 rpm, 5 min). After centrifugation twice, the supernatant was removed. The cells were resuspended in 300 µL of 4:1 diluted binding buffer, and transferred to different flow tubes for another 24 h-incubation. The cells were harvested and then incubated with PI and Annexin V-FITC using an apoptosis detection kit (KGA107, USA) following the instructions. Finally, cells were tested in flow cytometry (Becton Dickinson, FACS Verse, USA).

### Cell cycle experiments

Cells were seeded in six-well plates and grown overnight, which were treated with the different doses of AP probe-net (5, 20 µM) for 24 h. Next, the cells were digested with pancreatic enzyme without EDTA for 1 ~ 2 min and collected in the corresponding centrifuge tubes by a centrifugation (1000 rpm, 5 min). After centrifugation, 300 µL PBS was added to resuspend cells, followed by the addition of 700 µL pre-cooled anhydrous ethanol drop by drop, and incubated at 4℃ for 18–24 h. Then, the cells were added with 100 µL Rnase A solution and incubated in 37℃ water bath for 30 min. Finally, 400 µL of PI was added for staining of 30 min at room temperature in the dark. The cells were filtered using a 200-micron mesh before loading sample and further analysis.

### γ-H2AX test

The cells were inoculated in confocal culture dishes and treated with the increased doses of AP probe-net (5, 20 µM) for 24 h. First, the cells were fixed by adding 4% paraformaldehyde for 15 min. Next, 1 mL Triton-X-100(0.2%) and 1 mL 1% BSA were added respectively. The cells were stained withγ-H2AX antibody and cultured at 4℃ overnight. The antibody was recovered and then washed for 5 times. 100 µL of secondary antibody was added and incubated at room temperature for 1 h in the dark. Then, 1 drop of anti-fluorescence quenched-sealing tablet containing DAPI was added and stored at 4℃ in the dark. Finally, immunofluorescence analysis of the cells was performed using confocal microscopy (Axio-lmager-LSM-800 Germany).

### WB assay

Equal amounts of protein were separated by SDS-PAGE and then transferred onto nitrocellulose membranes. Membranes were blocked with tris-buffered saline tween (TBST) at room temperature for 60 min. After washing three times with TBST, membranes were incubated with primary antibodies at 4℃ overnight. Next, it was incubated with horseradish peroxidase-conjugated species-specific secondary antibody for 1 h and then washed for three times before further analysis.

### ATC tumor xenograft model

In this study, SPF BALB/C male nude mice aged 4 to 5 weeks, which were healthy and weighed within the normal range (15 to 18 g), were purchased from the Beijing Vital River Laboratory Animal Technology Co., Ltd. (Beijing, China). The feeding environment was a SPF-grade sterile laminar flow room with constant humidity (50–60%) and constant temperature (22–25℃). The food and drinking water were sterilized. The animal experiments were approved by the Animal Ethics Committee of Tianjin Medical University and Welfare Committee.

Next, we assessed the anti-tumor activity of AP probe-net in vivo, using nude mice for a xenograft study. CAL-62 cells (2 × 10^7^/mL) were subcutaneously implanted into the hind leg of each nude mouse until the tumors reached 7 mm^3^. After labeling nude mice, the formation of subcutaneous tumor masses could be observed about 10 days after inoculation, and the changes of tumor size were observed and photographed. Starting from the first day of tumor formation, the length (L) and width (W) of the tumor were measured with vernier calipers every 2 days to calculate tumor size. The tumor volume was calculated as follows:

V (mm^3^) = (L×W^2^) / 2

### AP probe-net treatment and pathological detection

Before treatment, the weight of each mouse and the size of the tumor were measured, and each mouse was photographed. The mice were randomized into 3 groups (3 mice per group) when the size of tumors reached to 7 mm^3^. The mice were then intraperitoneally injected with either AP probe-net (0.025 or 0.05 mg/kg/d with a volume of 500 µl every two days) or dimethyl sulfoxide (DMSO) for the negative control. The treatment of AP probe-net was discontinued after five injections and observed continuously for 24 days.

From the beginning of the first treatment, all mice were weighed and tumor size was measured with vernier calipers every 2 days. The tumor volume was calculated according to the above formula. In addition, tumors were photographed at day 0, 3, 6, 12, and 24 after the start of the trial. With the treatment time as the horizontal coordinate, the tumor volume and the mouse body weight as the vertical coordinate, the tumor growth curve and the mouse body weight change curve were plotted respectively.

After the end of treatment, all mice were anesthetized and injected intravenously [Equipment: PATTERSON VETERINARY Animal Anesthesia Machine. Chemical agents: isoflurane (2–4%)]. At the end of the experiment, all mice were sacrificed by cervical dislocation. The tumors were collected, photographed, weighed, and processed for paraffin sections. The heart, liver, lung, kidney and tumor tissues of each mouse were completely extracted and weighed. The tumor blocks were arranged according to the group and photographed for records. After that, the above tissues were soaked in formal forest for pathological detection. The expression of the Bax, casepase-3, Bcl-2 and Ki-67 in tumor tissues were detected by immunohistochemistry.

## Results

### GSH was significantly overexpressed in thyroid cancer cell

**Fig. 1:**
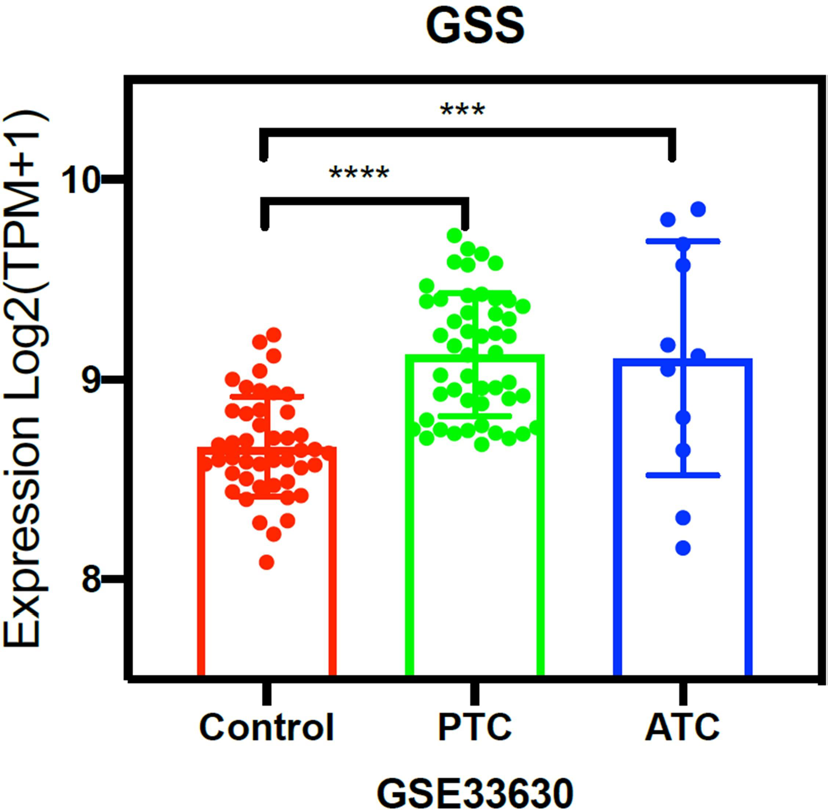
The expression levels of GSH enzyme (GSS) in PTC, ATC cells (cancer) and normal thyroid cells (normal). The data were retrieved from the Cancer Genome Atlas (TCGA) database. (* *P* < 0.05, ** *P* < 0.01, *** *P* < 0.001, **** *P* < 0.0001)

**Fig. 2:**
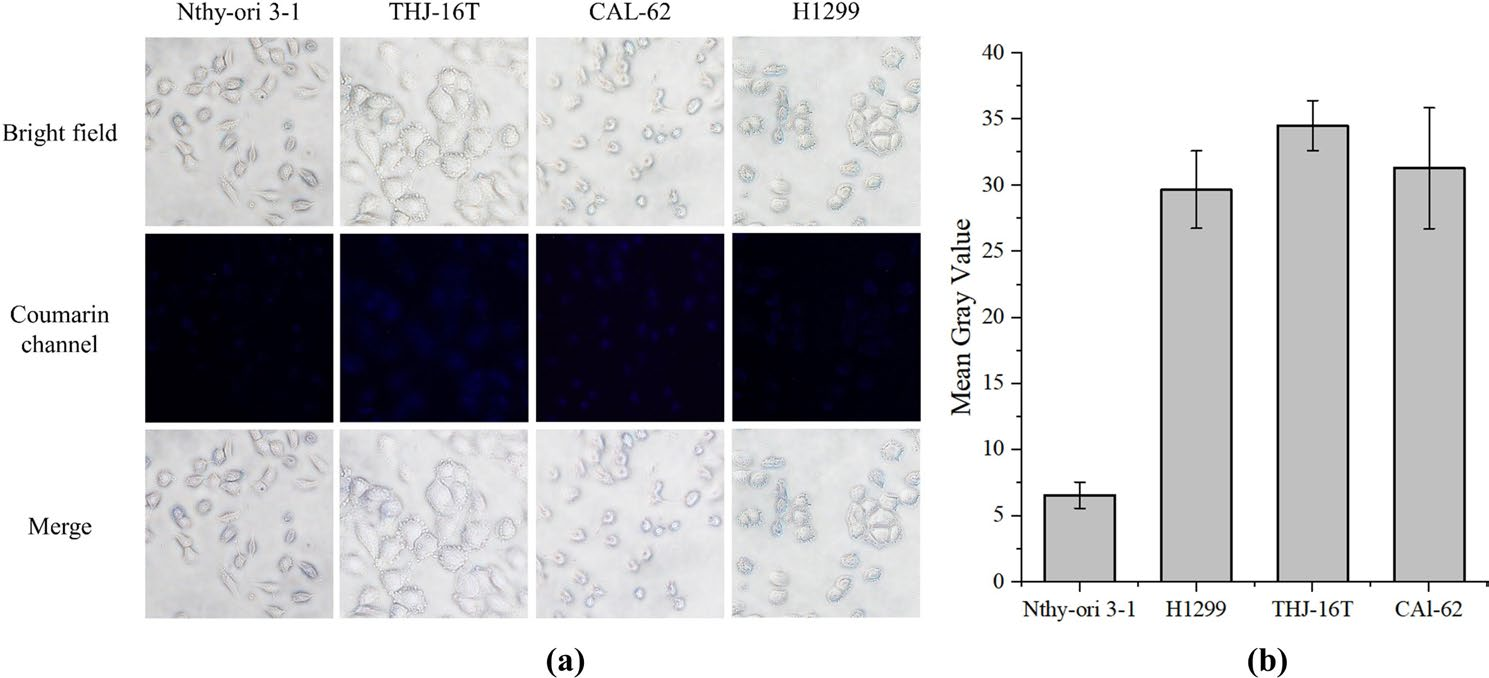
Determining GSH levels in normal thyroid cells (Nthy-ori 3 − 1), lung cancer cells (H1299), and ATC cells (THJ-16T and CAL-62) using AP probe-net. The cells were incubated with AP probe-net (10 µM, 1 h) and washed with buffers before test. Data are presented as mean ± standard deviation

According to data retrieved from the Cancer Genome Atlas (TCGA) database, the glutathione enzyme (GSS) expression levels in thyroid cancer cells were significantly higher than that in normal thyroid cells (Fig. 1). We also checked the GSH levels in these cells using the AP probe-net, which is non-fluorescent, but can restore strong fluorescence upon the reaction with cellular GSH. As shown in the Fig. 2, the fluorescence intensity of the two ATC cancer cells (THJ-16T and CAL-62) were comparable to that of lung cancer cells (H1299), but significantly 4 times higher than that of normal thyroid cells (Nthy-ori 3 − 1), indicating that the concentration of GSH in ATC cells was significantly higher than that of normal thyroid cells. GSH responsive AP probe-net may show selective anti-tumor activity towards ATC cancer cells over normal thyroid cells due to their elevated GSH levels (Synthetic route of AP probe-net was shown in the Supplementary Fig. 1). It should be noted that the cancer cells with abundant GSH also contain a high concentration of H_2_O_2_, resulting in DNA oxidative damage [10]. It will produce 8-oxo-guanines and promote action of OGG1 to generate AP sites. As such, increased GSH levels would correlate with higher levels of AP sites, facilitating the potent and selective cytotoxicity of GSH responsive AP probe-net in ATC cells. Previously, selective antitumor activity was observed in H1299 lung cancer cells over WI38 normal lung fibroblasts cells, and the principle and mechanism of GSH-responsive AP probe-net had been briefly confirmed in vitro [11]. Whether this strategy can be employed in treating ATC in vitro and in vivo is crucial and meaningful.

### Cytotoxicity studies

**Fig. 3:**
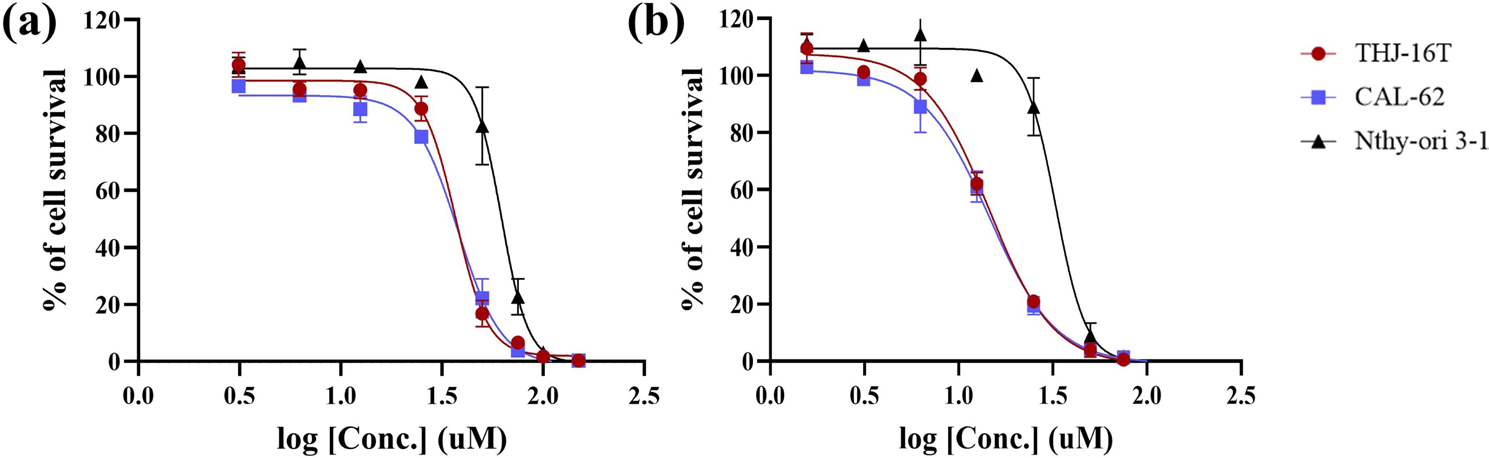
Cytotoxicity of AP probe-net in ATC cells (THJ-16T and CAL-62) and normal thyroid cells (Nthy-ori 3 − 1) for 24 h (**a**) and 48 h (**b**). Representative IC_50_-curve from three different experiments is shown

The elevated levels of GSH in ATC cells could promote the release of reactive alkoxyamine to trap AP sites, block DNA damage repair, and induce ATC cell death. In this way, they would exhibit targeted anti-tumor activities. To confirm this hypothesis, we conducted cytotoxicity assays, and half-maximal inhibitory concentration (IC_50_) values of the agents against ATC cells and normal thyroid cells were determined using the standard MTT assay (Fig. 3; Table 1). All cell lines were sensitive to AP probe-net upon incubation for either 24–48 h, but stronger cytotoxicity was observed in ATC cells (IC_50_ = 14.5 µM for THJ-16T; IC_50_ = 13.5 µM for CAL-62) over normal thyroid cells (IC_50_ = 32.7 µM) upon incubation for 48 h (Table 1). There was a similar trend upon incubation for 24 h. Both revealed that AP probe-net displayed selective cytotoxicity towards ATC cells over normal thyroid cells.

**Table 1:**
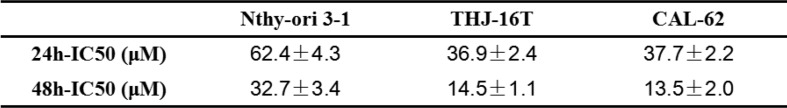
IC_50_ of AP probe-net in ATC cells (THJ-16T and CAL-62) and normal thyroid cells (Nthy-ori 3 − 1) at 24 h and 48 h. Data are presented as mean ± standard deviation of triplicate experiments

### Apoptosis and cell cycle analysis

**Fig. 4:**
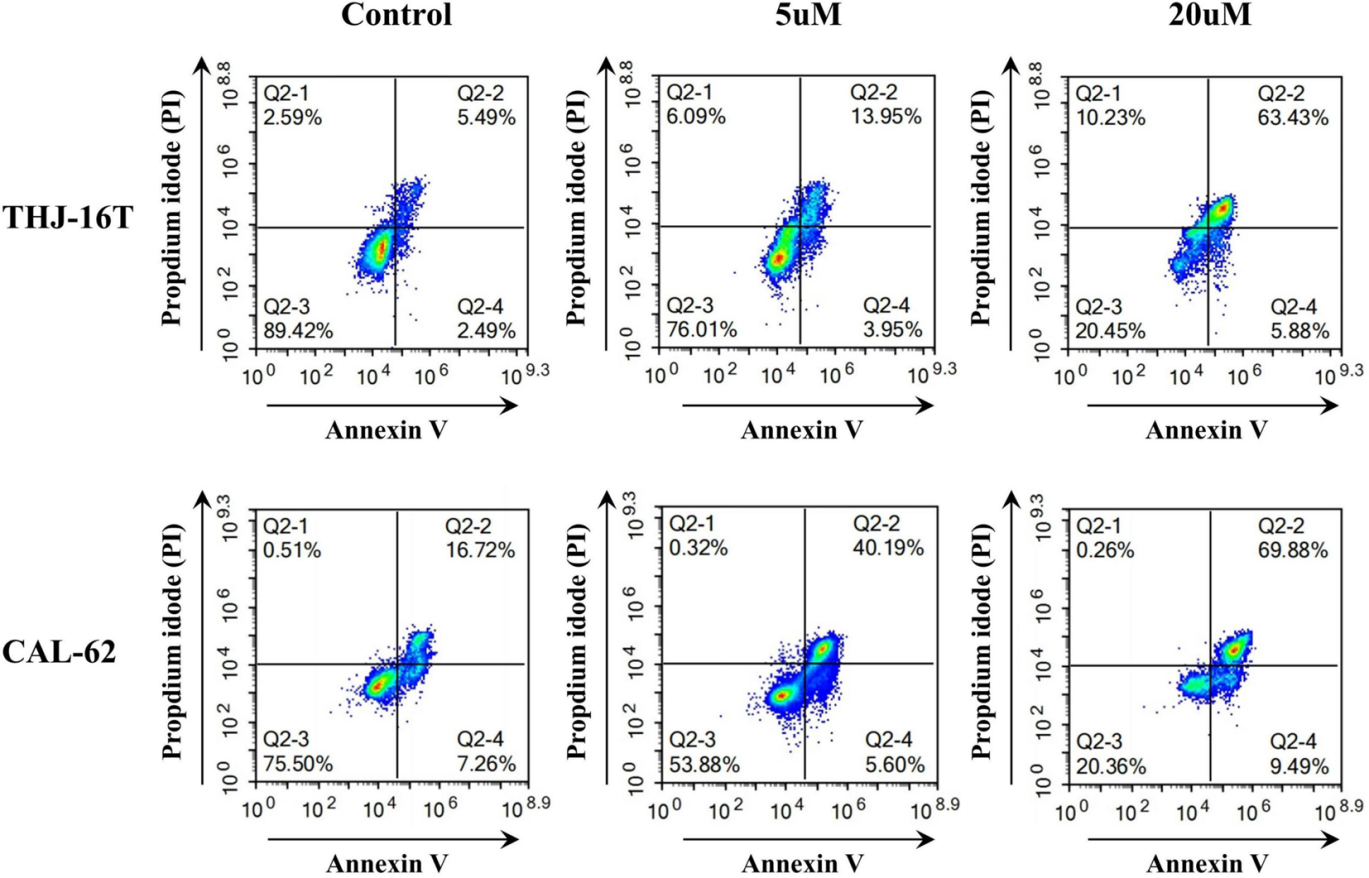
Annexin V/PI staining and flow cytometry analysis of THJ-16T and CAL-62 cells with different doses of AP probe-net treatment for 24 h. (Q2-3: Annexin V negative and PI negative populations represent healthy cells. Q2-4: Annexin V positive and PI negative populations represent cells in early apoptosis. Q2-2: Annexin V positive and PI positive populations represent cells in late apoptosis)

To further facilitate our understanding about their cytotoxicity towards ATC cells, we also analyzed the cellular responses evoked by the AP probe-net using multiparameter flow cytometry (Fig. 4). We focused on its effects on apoptosis and cell cycle. A dual Annexin V staining/PI flow cytometry assay revealed that AP probe-net significantly enhanced apoptosis in the late stage (Annexin V+/PI + channel), which can be stained by both Annexin V and PI. The percentage of late apoptosis in THJ-16 cells increased from 5.49% for untreated cells to 63.43% for treated cells, while the corresponding percentage in CAL-62 cells increased from 16.72 to 69.88%. With the increased doses of AP probe-net, the percentage of apoptosis also increased.

**Fig. 5:**
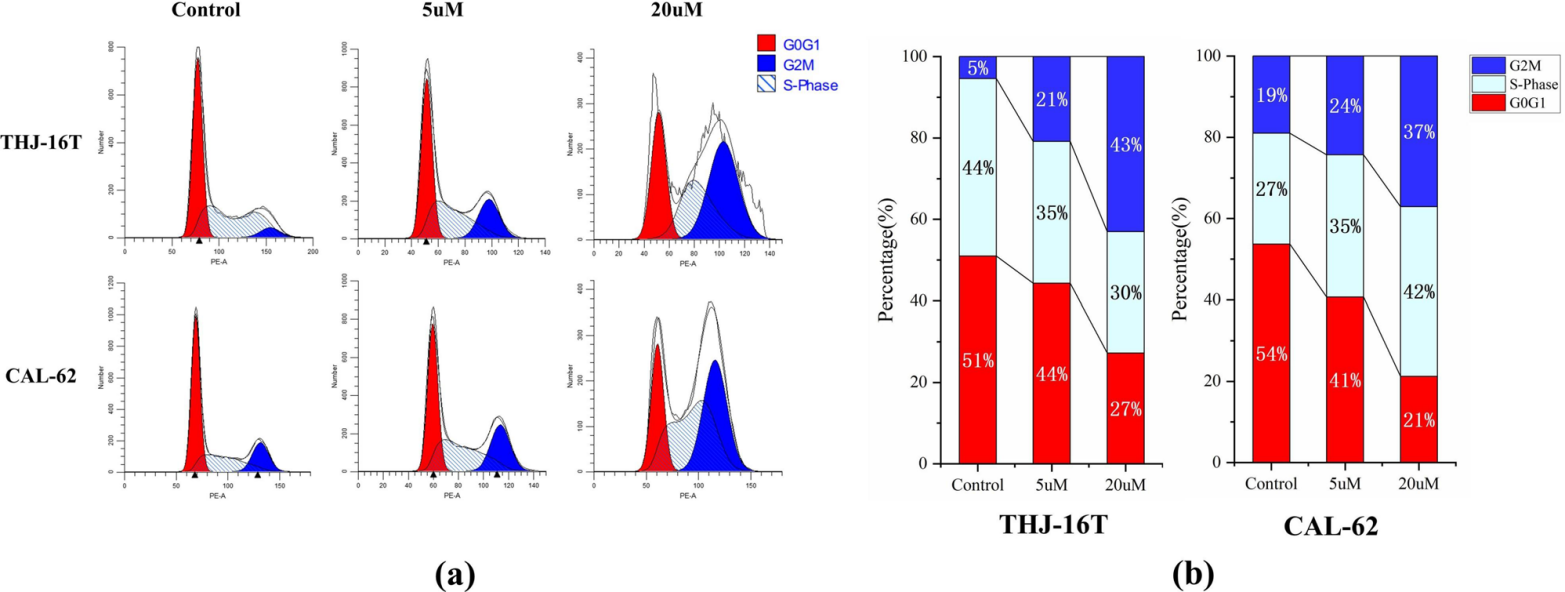
Cell-cycle analysis in ATC cells (THJ-16T and CAL-62) with different AP probe-net doses. (**a**) Cell cycle analysis via flow cytometry upon propidium iodide staining; (**b**) Cell distribution patterns in the three major phases of the cell cycle using DNA-content analysis

Cell-cycle analysis indicated that AP probe-net arrested the cell cycle in the G2/M phase both in THJ-16T and CAL-62 cells (Fig. 5). As the concentration of AP probe-net increased, the proportion of cells in G2/M phase gradually increased. For example, the percentage of cells in G2/M phase increased from 5% for the control to 43% for THJ-16t upon incubation with AP probe-net (20 µM). Accordingly, the proportion of cells in G0/G1 phase gradually decreased from 51 to 27%. Similar trend was observed in CAL-62 cells.

### DNA strand breaks induced apoptosis

**Fig. 6:**
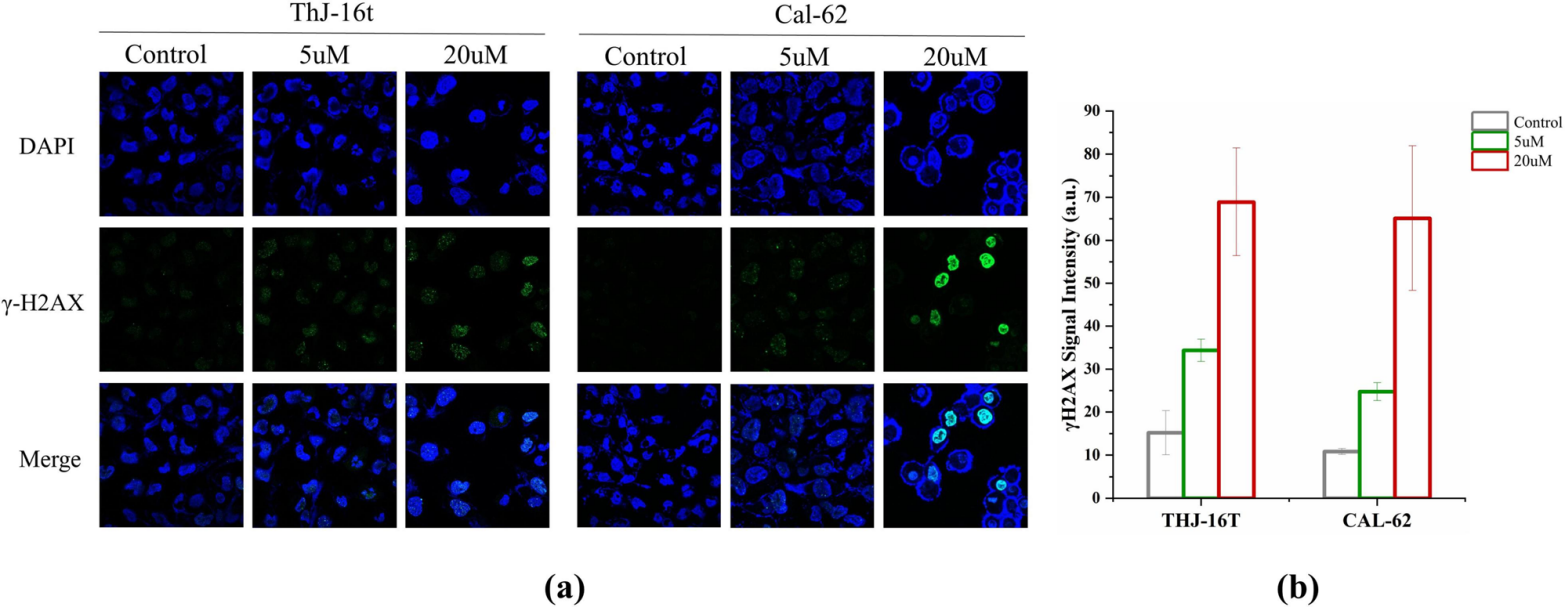
γ-H2AX levels in ATC cells (THJ-16T and CAL-62) with different AP probe-net doses. (**a**) Confocal fluorescence microscopy images of DAPI (blue) staining nuclei and γH2AX (green) staining in cells; (**b**) Quantification of γH2AX signal intensity using Image J. Data are presented as mean ± standard deviation

**Fig. 7:**
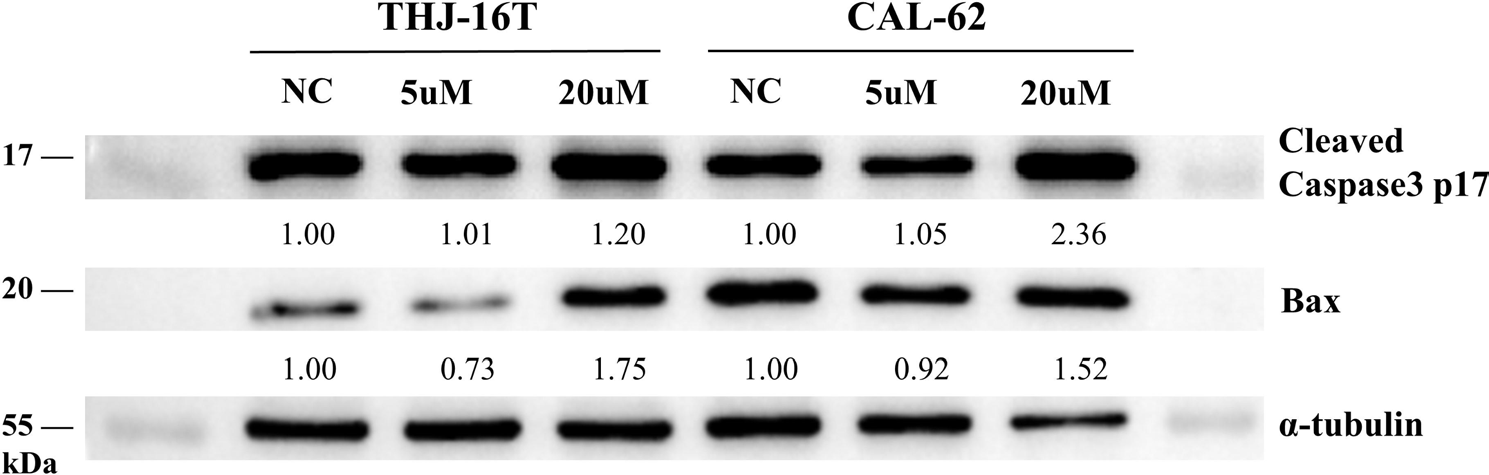
Levels of cleaved Caspase-3 and Bax assessed by western blotting for the cells treated with AP probe-net for 12 h. Cleaved caspase-3 and Bax were both upregulated in ATC cells treated with AP probe-net. The WB gels of Fig. 7 were the cropping of the full-length gels. The full-length gels are presented in Supplementary Fig. 2

G2/M phase arrest and apoptosis often result from DNA damage in chemotherapy. The AP trapping product by AP probe-net is also one form of DNA lesions, inducing further DNA damage. To test this hypothesis, we first carried out a γ-H2AX assay to detect double-strand breaks (DSBs) in cells [12]. DSB detection was based on expression levels of γ-H2AX, a classic biomarker for DSBs, which could be visualized by immunofluorescence microscopy. A significantly enhanced γ-H2AX fluorescence intensity was observed in cells upon treatment with AP probe-net (5 µM and 20 µM). These results suggested that AP probe-net induced the accumulation of DNA damage and DNA strand breaks in both THJ-16T and CAL-62 cells (Fig. 6). Western blotting assay was used to detect whether AP probe-net alters the expression levels of proapoptotic proteins in THJ-16T and CAL-62 cells. The cleaved casepase-3 and Bax played a vital role in the regulation of apoptosis. Our data showed that cleaved casepase-3 and Bax were both upregulated in THJ-16T and CAL-62 cells upon treatment with AP probe-net (20 µM) for 12 h (Fig. 7). These results collectively indicated that the treatment with AP probe-net potently induced apoptosis in ATC cells.

### AP probe-net treatment and pathological detection

**Fig. 8:**
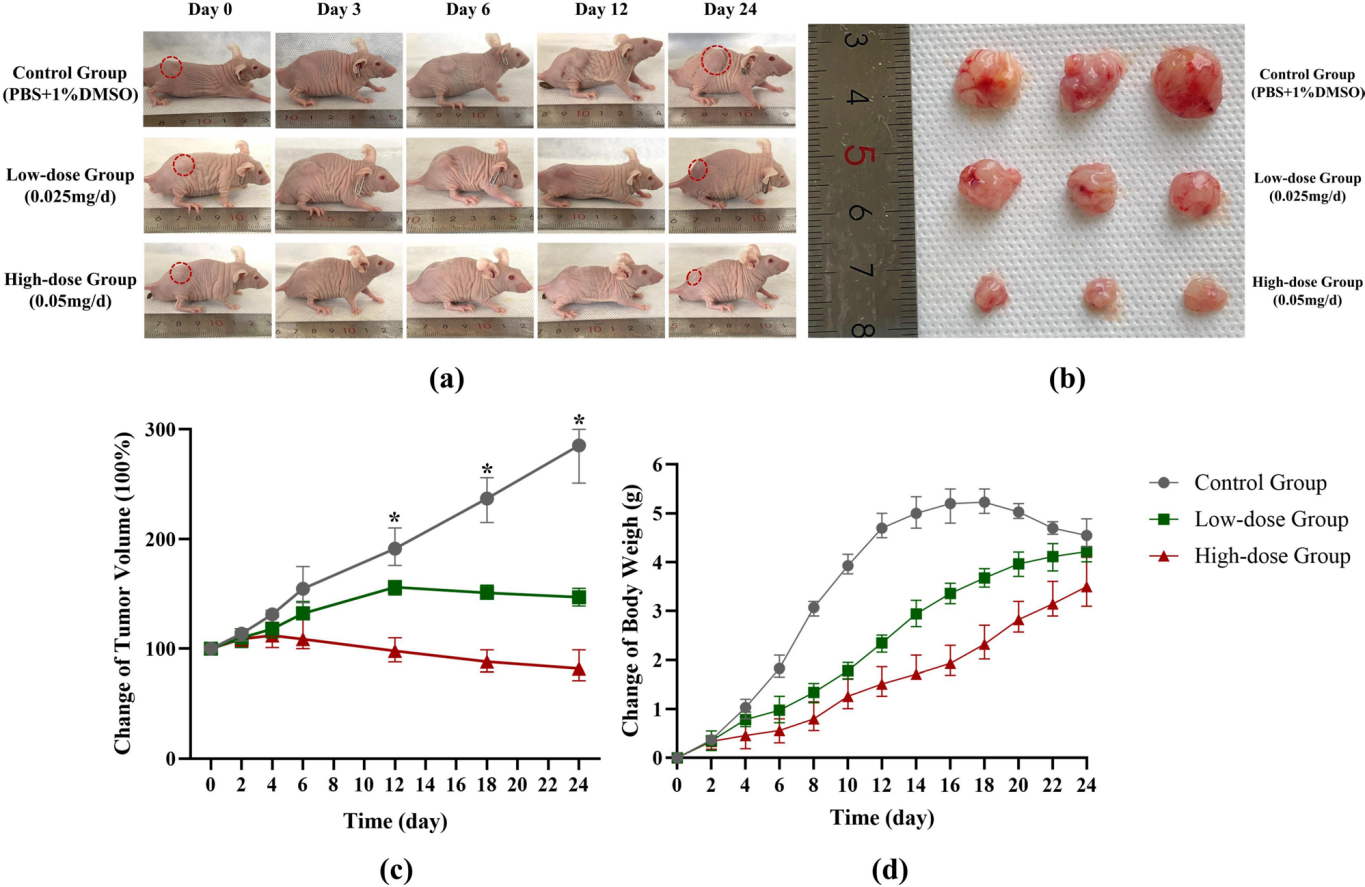
In vivo study of AP probe-net against ATC. (**a**): Representative photos of mice before (0 days) and after 3-, 6-, 12-, and 24-days of treatment. (**b**): Pictures of the excised tumors after different treatments for 24 days. (**c**): Changes of tumor volume from subgroups (* *P* < 0.05). (**d**): Changes of the mice body weights from subgroups

**Fig. 9:**
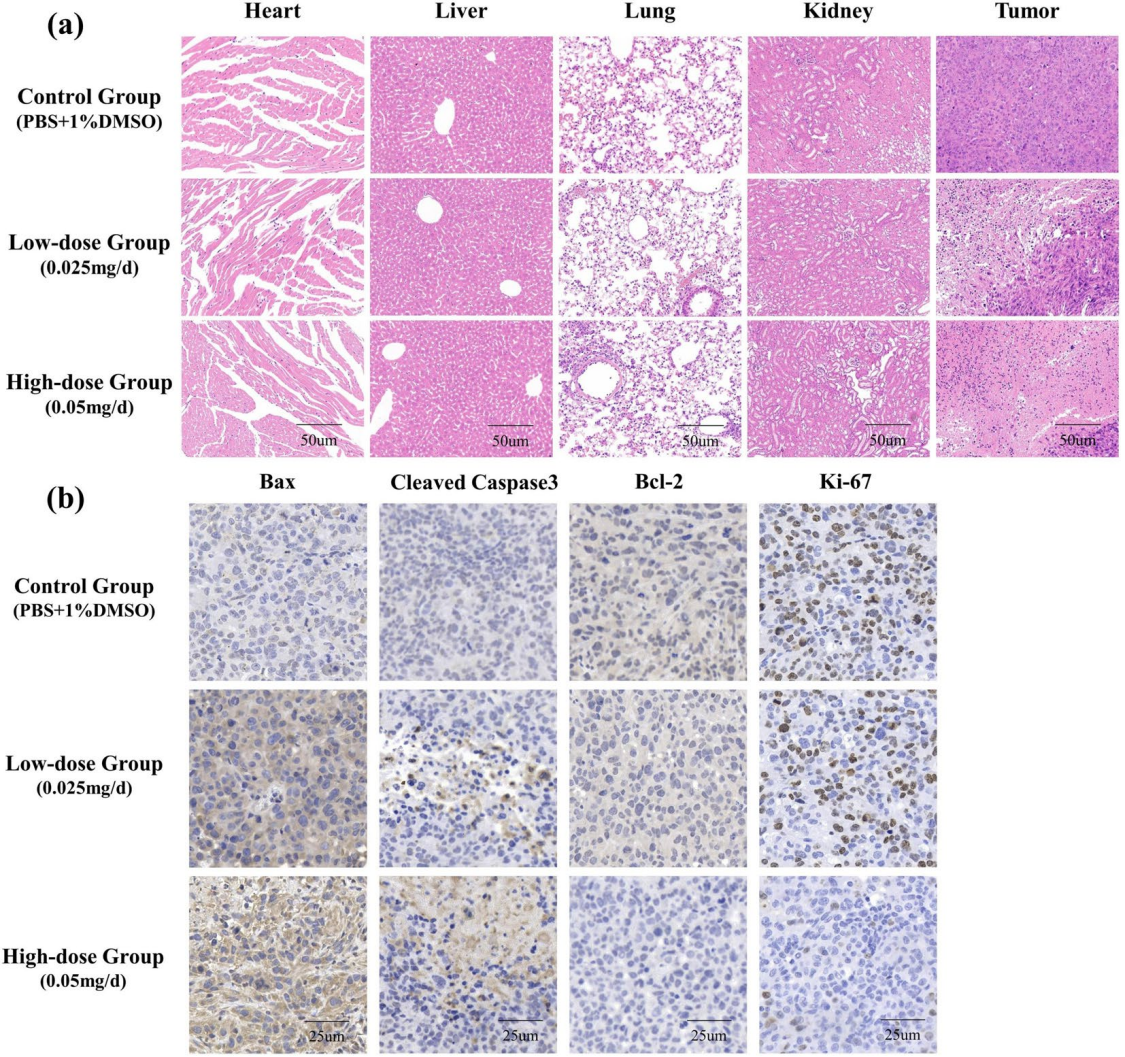
The toxicity towards organisms and the immunohistochemical assay. (**a**) Hematoxylin and Eosin Staining of tissues and tumor sections of mice upon different treatments (the scale bar = 50 μm). (**b**) Immunohistochemistry analysis of Bax, Cleaved-Caspase-3, Bcl-2 and Ki67 in tumor sections (the scale bar = 25 μm)

Our data showed that the suppressed tumor growth in a dose-dependent manner in vivo, with both the decreased final tumor volumes and weights following AP probe-net treatment (Fig. 8). No obvious toxicity was observed in any of the treatment groups, based on the average body weight, and the size and morphology of heart, liver, lung and kidney tissues, which did not differ significantly from that of control mice (Fig. 9). These results suggested that AP probe-net could suppress xenograft tumor growth in vivo without obvious adverse effects. Immunohistochemical of these tumor cells were shown in Fig. 9. Immunohistochemical data of tumor tissue and previous WB results consistently showed that Bcl-2 and Ki-67 were down-regulated, while cleaved casepase-3 and Bax were up-regulated in a dose-dependent manner (Fig. 9).

## Discussion

There are approximately 10^12^ to 10^13^ cells in the human body that coordinate with each other to maintain the homeostasis of the body [13]. However, many endogenous and exogenous factors such as free radicals produced by chronic inflammation in the body, certain metabolites of cells and gut microbiota [14], as well as chemical carcinogens in the environment [15], ultraviolet (UV) radiation attack intracellular DNA and may cause a variety of DNA damage [3]. If these DNA damage are not repaired correctly and timely, it will pose a threat to the stability and integrity of biological genomes, and even affect the normal life activities of cells or the body [16]. This often occurs in chemotherapy. Among the induced DNA lesions, abasic (AP) sites are one of the most common types of formed damage in cells from the depurination/depyrimidination of damaged bases or as intermediates in the BER pathway. It is generated by the specific excision of n-β-glucoside bonds on damaged nucleotides by the nucleoside hydrolase in the cell [17, 18]. If AP sites are not repaired in time, they will affect the proper function of RNA polymerase and DNA polymerase. For example, DNA strand breaks and reversible cross-links with DNA or histone proteins of nucleosome core particles from AP sites affect transcription and replication in cells, further inducing gene mutations, which may cause apoptosis or cell carcinogenesis [19, 20]. In addition, recent chemotherapy studies suggested that AP trapping products blocked AP site repair and induced cell death. Thus, trapping AP sites and blocking their repair by APE1 will result in cytotoxicity.

APE1 plays an irreplaceable role in both BER and NER pathways [21]. It functions in oxidative and alkylated genomic DNA base repair by identifying and cutting nucleotide chains at the 5’apurinic (AP) site. In mammalian cells, APE1 also has a unique REDOX function, directing the activity of different transcription factors, thereby affecting gene expression and protein production [22]. Codrich, et al. found that the DNA repair and REDOX functions of APE1 were also essential for maintaining mitochondrial integrity [23]. APE1 was also one of the many non-regulatory proteins involved in cancer survival signaling pathways, and could be used as a therapeutic target in the treatment of multiple types of tumor cells [24]. Studies had found that APE1 was overexpressed to repair DNA damage in non-small cell lung cancer (NSCLC) tumor tissues, and patients with low APE1 levels significantly increased progression-free survival and overall survival [25]. Fishel, et al. revealed that the use of a novel REDOX selective APE1 inhibitor in vitro and in vivo could effectively reduce the progression of bladder cancer, and the combination of this inhibitor with cisplatin, a commonly used chemotherapy drug, was more effective than cisplatin alone [26]. APE1 and its regulatory factor NPM1 could also reduce the cytotoxicity of platinum-based chemotherapy drugs to triple-negative breast cancer cells, and reduced levels of APE1 in breast cancer cells could improve its sensitivity to olapalil [27]. APE1 inhibitor APX3330(E3330) could promote the treatment of colorectal cancer tumors by inhibiting the REDOX function of APE1 in combination with 5-FU. However, inhibition of endonuclease activity of APE1, rather than REDOX function, could interact with NPM1 to trigger p53-mediated effects on colon cancer cell metabolism and improve its sensitivity to targeted drugs [22]. Numerous studies had demonstrated that APX3330 specifically inhibited APE1 REDOX signaling in a variety of tumor models in vitro and in vivo, including pancreatic cancer, leukemia, and malignant peripheral nerve sheath tumors (MPNSTs) [28]. In glioblastoma (GBM) patients, oxidative stress leaded to elevated APE1 levels, which increased repair of the AP sites from the chemotherapy, leading to increased drug resistance. The REDOX and repair functions of APE1 were also associated with temozolomide (TMZ) resistance [29]. The inhibition of APE1 expression or activity impacted the cell cycle, cell proliferation, colony formation, and apoptosis of cancer cells [30, 31]. For these reasons, APE1 was considered to be a promising prognostic cancer biomarker and therapeutic target. Our preliminary research developed a GSH responsive AP capture reagent [11], which were selectively activated by GSH to produce reactive alkoxyamines (AP capture agents). It was more effectively activated by elevated levels of GSH in H1299 lung cancer cells than that in normal lung fibroblasts WI38 cells, releasing reactive alkoxyamines to capture AP sites, blocking APE1 repair, and leading to lung cancer cell death in vitro. No studies had been reported on trapping AP sites and inhibiting APEI-mediated AP sites repair for the treatment of thyroid cancer.

In addition, a large number of anti-tumor drugs were reported to damage DNA, produce AP sites and promote apoptosis, and AP capture efficiently enhanced their cytotoxicity [32]. However, a little attention has been paid to reducing the side effects on normal cells from unselective AP capture, limiting their future development. Usually, aldehyde-reactive alkoxyamines captured AP sites and blocked the activity of APE1 without selectivity towards different cells, causing side effects towards normal cells. The selective release of alkoxyamines by an abundant biomolecule in cancer cells offers a solution for this challenge. Targeted anti-tumor agents that take advantage of the unique chemical environments of cancer cells greatly reduced side effects on normal cells. Considering the abundance of GSH in ATC cells, we developed GSH-responsive AP trapping agents with targeted anti-tumor activity. GSH rapidly activates DNS-caged alkoxyamines, producing SO_2_ and fluorescent alkoxyamines that trap AP sites in live cells. Reports have revealed that the releasing SO_2_ may disrupt the cellular redox equilibrium, and show cytotoxicity to some extent [33]. And the GSH-responsive AP probe-net captures AP sites, and block the APE1 activity, inducing cell death. Importantly, selective toxicity of the agent against ATC cells over normal thyroid cells were observed. This was attributed to the greater GSH concentration in the former. Cellular studies, including cytotoxicity studies, and the DNA damage test, demonstrated the essential roles of AP probe-net in their cytotoxicity. Flow cytometry analysis suggested that AP probe-net arrested the cell cycle in the G2/M phase and induced apoptosis of cells. The better therapeutic efficacy of AP probe-net was evaluated in an ATC cell line subcutaneous tumor. The pathological results showed no observed damage to the liver, kidney and other important organs of mice, and the treatment effect of ATC tumor was significant. The results of tumor immunohistochemistry further confirmed the results of WB, which caused apoptosis. Overall, our research on GSH-responsive AP probe-net affords a new strategy for targeted chemotherapy in ATC.

## Conclusions

Taken together, these findings suggested that GSH-responsive AP probe-net could selectively suppress ATC cell growth in vitro as well as xenograft tumor growth in nude mice, and induce less adverse effects towards normal cells or tissues. GSH-responsive AP probe-net, responding to elevated GSH in ATC cells, triggered the DNA double-strand breaks, promoted apoptosis, inhibited cell proliferation, and induced ATC cells death with high selectivity and reduced adverse effects. Overall, our study on GSH-responsive AP probe-net afforded a new strategy for targeted chemotherapy in ATC treatment.

### Electronic supplementary material

Below is the link to the electronic supplementary material.

Supplementary Material 1

Supplementary Material 2

## Data Availability

The data that support the findings of this study are available upon reasonable request from the corresponding author.

## Abbreviations

ATC: anaplastic thyroid carcinoma
AP: apurinic/apyrimidinic or abasic
APE1: apurinic/apyrimidinic endonuclease 1
BER: base excision repair
GSH: glutathione
TME: tumor micro-environment
AP probe-net: AP sites capture reagent
WB: western blotting
RIT: radioiodine therapy
DNS: 2,4-dinitrobenzene sulfonamides
MTT: 3-(4,5-dimethylthiazol-2-yl)-2,5-diphenyltetrazolium bromide
RMPI 1640: Roswell park memorial institute 1640
FBS: fetal bovine serum
DMSO: dimethyl sulfoxide
TBST: tris-buffered saline Tween
GSS: glutathione enzyme
DSBs: double-strand breaks
NSCLC: non-small cell lung cancer

## Declarations

### Animal studies

The Registration No. of the study was IR B2022-DWFL-009.
