# Supplementary material for: Selective anti-tumor activity of glutathione-responsive abasic site trapping agent in anaplastic thyroid carcinoma
Source: BMC Cancer. 2024 Jul 8;24:816. doi: 10.1186/s12885-024-12511-3 (PMC11229194; doi:10.1186/s12885-024-12511-3)
Supplement: Supplementary file 2 — Supplementary Material 2 [file 12885_2024_12511_MOESM2_ESM.docx]

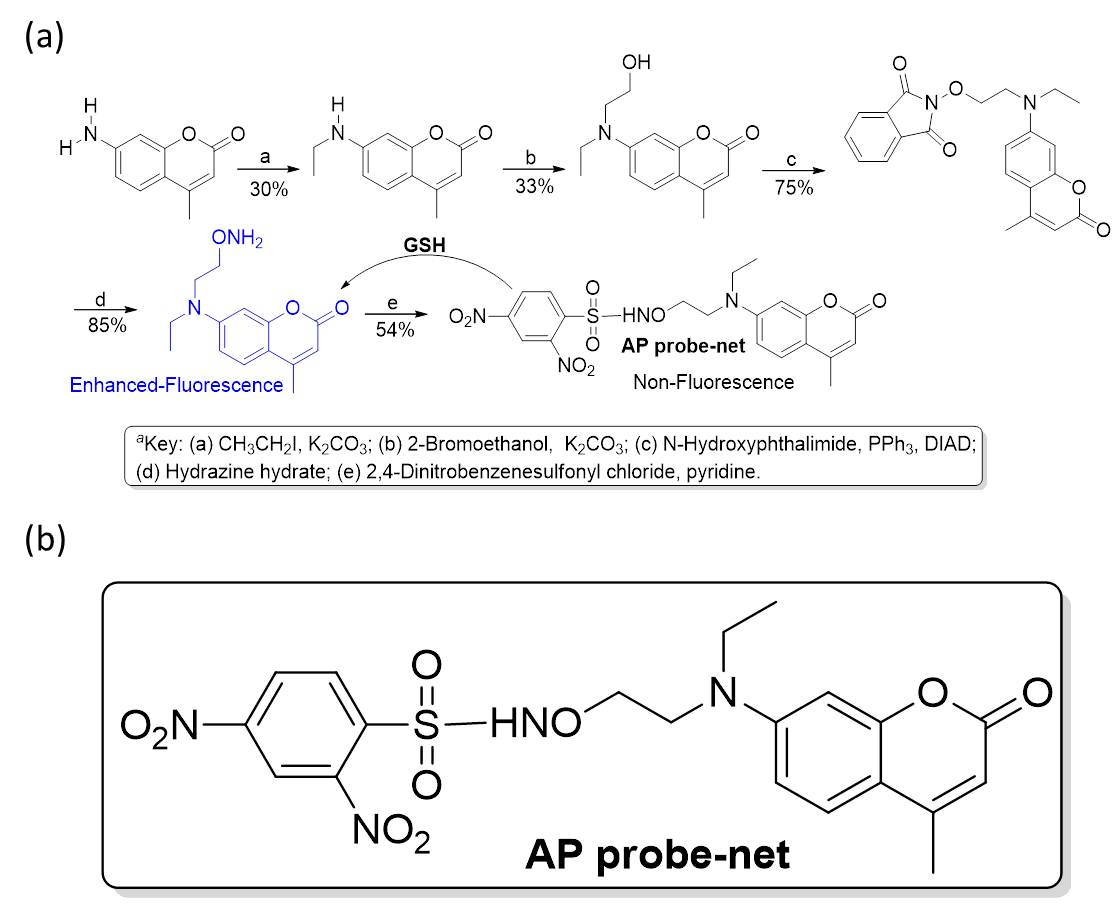


Supplementary Figure 1. The synthetic route of AP probe-net (a) and the structure of AP probe-net (b).


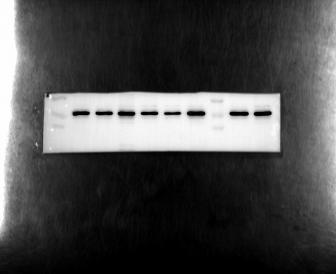

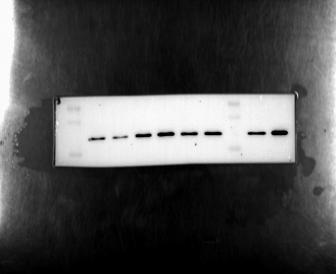

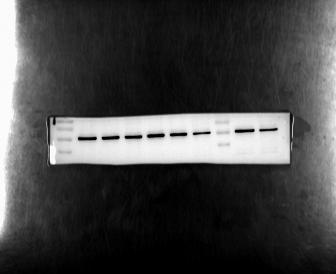


**Cleaved Caspase-3 p17**

**Bax**

**α-tubulin**

(a)

(b)


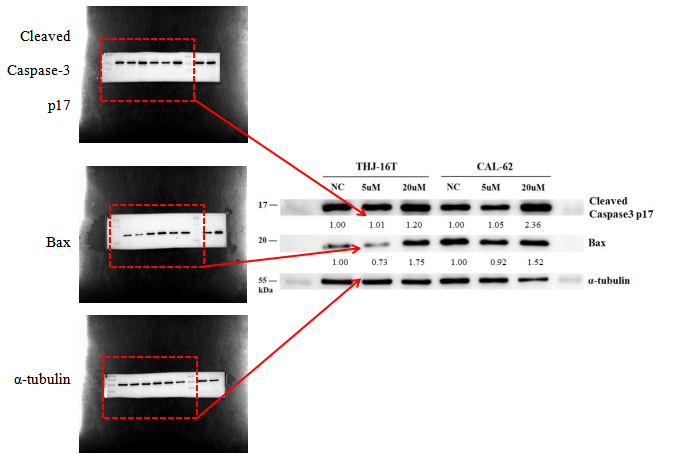


Supplementary Figure 2. (a): The full uncropped Gels of cleaved casepase-3, Bax and α-tubulin. (b): A summary graph about the original images and the figure legends matched with Figure 7.
